# The mental health of neurological doctors and nurses in Hunan Province, China during the initial stages of the COVID-19 outbreak

**DOI:** 10.1186/s12888-020-02838-z

**Published:** 2020-09-05

**Authors:** Xianjun Ning, Fang Yu, Qin Huang, Xi Li, Yunfang Luo, Qing Huang, Changqing Chen

**Affiliations:** 1 Department of Neurology, Xiangya Hospital, Central South University, Changsha, Hunan 410008 PR China; 2 Teaching and Research Section of Clinical Nursing, Xiangya Hospital of Central South University, Changsha, PR China

**Keywords:** COVID-19, Anxiety, Depression, Neurology, Healthcare workers

## Abstract

**Background:**

Neurological symptoms are increasingly being noted among COVID-19 patients. Currently, there is little data on the mental health of neurological healthcare workers. The aim of this study was to identify the prevalence and influencing factors on anxiety and depression in neurological healthcare workers in Hunan Province, China during the early stage of the Coronavirus Disease 2019 (COVID-19) outbreak.

**Methods:**

An online cross-sectional study was conducted among neurological doctors and nurses in early February 2020 in Hunan Province. Symptoms of anxiety and depression were assessed by the Chinese version of the Self-Rating Anxiety Scale (SAS) (defined as a total score ≥ 50) and Self-Rating Depression Scale (SDS) (defined as a total score ≥ 53). The prevalences of probable anxiety and depression were compared between different groups, and multivariate logistic regression analysis was used to understand the independent influencing factors on anxiety and depression.

**Results:**

The prevalence of probable anxiety and depression in neurological nurses (20.3 and 30.2%, respectively) was higher than that in doctors (12.6 and 20.2%, respectively). Female healthcare workers (18.4%) had a higher proportion of anxiety than males (10.8%). Probable anxiety and depression were more prevalent among nurses, younger workers (≤ 40 years), and medical staff with junior titles. Logistic regression analysis showed that a shortage of protective equipment was independently associated with probable anxiety (OR = 1.980, 95% CI: 1.241–3.160, *P* = 0.004), while young age was a risk factor for probable depression (OR = 2.293, 95% CI: 1.137–4.623, *P* = 0.020) among neurological healthcare workers.

**Conclusions:**

Probable anxiety and depression were more prevalent among neurological nurses than doctors in Hunan Province. The shortage of protective equipment led to probable anxiety, and young age led to probable depression in healthcare workers in neurology departments, which merits attention during the battle against COVID-19.

## Background

After first emerging in Wuhan, China in December 2019, the 2019 novel coronavirus (2019-nCoV), now dubbed COVID-19, quickly spread throughout the world [1]. This new coronavirus has turned out to be much more infectious than severe acute respiratory syndrome coronavirus (SARS-CoV) and Middle East respiratory syndrome coronavirus (MERS-CoV), which caused massive outbreaks [2, 3].

Many clinical studies of the epidemiological and clinical characteristics of COVID-19 cases have been published [4–6]. Unlike SARS, which mostly affected young and middle-aged people, the novel coronavirus tends to result in serious illness in elderly people, especially those with chronic diseases, such as diabetes, hypertension, and cardiovascular and cerebrovascular diseases [6, 7]. The main initial clinical manifestations include fever, cough, and shortness of breath. However, some patients do not have major symptoms, developing atypical initial symptoms in the digestive system or nervous system, which makes proper diagnosis difficult [8]. Furthermore, a proportion of 2019-nCoV-positive patients, have nonspecific symptoms or even are asymptomatic, a state in which the virus can still spread and cause infection [9–11]. At present, our understanding of this novel coronavirus is incomplete. Respiratory droplets and person-to-person contact are defined routes of transmission for coronavirus infection, while transmission via the airborne, fecal-oral and aerosol routes remains ambiguous [12–14].

Since the outbreak, a shortage of medical supplies has been a weakness in the prevention and control of the epidemic and has severely restricted the treatment of patients and threatened the safety of medical staff [15]. An epidemiological study of the first 72,314 cases of COVID-19 identified on the Chinese mainland from December 31, 2019, to February 11, 2020 reported that at least 3000 medical workers had been infected with 2019-nCoV [1]. Medical protective supplies were mainly distributed to the designated COVID-19-accepting hospitals, intensive care units, and fever outpatient departments. Medical personnel in general hospitals and other departments outside the front-line may be at high risk of infection because they do not have enough personal protective equipment.

Some studies have reported that several respiratory viruses, such as SARS-CoV, have the capacity to spread from the respiratory system to the central nervous system (CNS), causing encephalitis and other neurological diseases [16]. Recent reports of COVID-19 patients in Wuhan described several neurological manifestations, including headache, myalgia, nausea, dizziness, taste and smell impairment, vision impairment and neuralgia [4, 6, 17]. Direct or indirect cardiotoxicity secondary to excessive systemic proinflammatory stimulation, hypercoagulability, myocardial infarction, heart failure, and arrhythmias are important risk factors for stroke in patients with COVID-19 [18]. From several studies reporting COVID-19 patients presenting with stroke, two patients had no COVID-19-related symptoms prior to the stroke [19, 20]. If a COVID-19 patient visits a neurology department with such neurologic symptoms as the initial symptom without fever or respiratory discomfort, he or she could be underdiagnosed and become a main spreader of infection. Medical workers could become infected through contact with those patients. Furthermore, a shortage of protective supplies and lack of training to care for patients with COVID-19 contributes to the high risk of infection among neurological health care workers. Unlike medical workers who are fighting on the front line, doctors and nurses in neurology departments may be less likely to receive training, including diagnostic testing for COVID-19, responses to infectious diseases, and appropriate donning and removal of personnel protective equipment [21].

Biological disasters have a high risk of spreading infection and increased mortality, which increases the fear of social contact, especially for health care staff who have close contact with infected patients during outbreaks [22]. It is believed that the prevention of mental health disorders should receive more attention to minimize the negative health impact of infectious diseases [23]. The experience with SARS indicated that the prevalence of psychiatric morbidities, such as fear and anxiety, was high among medical workers, patients, and even the general public [24, 25].

Studies are increasingly looking at the psychological impacts on the general population [14], psychiatric patients [26] and medical health workers [27], indicating that COVID-19 does have effects on the mental health of these populations to varying degrees. Currently, no studies have focused on the psychological impact on a particular group, such as neurological medical workers. Mental health care for medical workers, whether working on the front line or not, should receive serious consideration. Thus, the aim of this study was to investigate the mental health of medical workers in departments of neurology during the outbreak of COVID-19 in Hunan Province. Studying the psychological impact on neurology medical workers may provide valuable information for other out of front-line departments, such as ophthalmology and otolaryngology.

## Methods

### Participants

Because the investigation was performed during the COVID-19 epidemic, the quarantine measures demanded less face-to-face communication and contact. Therefore, self-administered anonymous electronic questionnaires were conducted on a non-commercial mobile app called “Questionnaire Star.” In early February 2020, we distributed those questionnaires to neurological healthcare workers in Hunan Province via WeChat, which is the most widely used social networking software in China, using a snowball sampling approach. Participants were encouraged to send the questionnaire link to their colleagues whom they considered suitable for this survey. Finally, effective questionnaires were returned from 650 medical workers, of which 612 were valid, including 317 doctors and 295 nurses. The protocol of this study was approved by the local Ethics Committee of Xiangya Hospital, and all participants completed the self-reported questionnaire after providing informed consent.

### Sociodemographic factors

Demographic characteristics of the participants, such as age, sex, educational level, occupational title and marital status, were collected in this study.

### Attitudes toward the epidemic

In the electronic questionnaire, the following three questions addressed respondents’ perceptions of COVID-19 during the outbreak: 1) Do you think the department of neurology is a high-risk place for COVID-19? 2) Are your current precautions adequate to prevent infection? 3) Are you willing to treat or care for patients with COVID-19?

### Assessment of anxiety and depression

We adopted the Chinese edition of Zung’s Self-Rating Depression Scale (SDS) and Zung’s Self-Rating Anxiety Scale (SAS) to assess probable anxiety and depression. The widely used SAS and SDS scales are quick and convenient scales to evaluate anxiety and depressive symptoms of investigated subjects and are valid and efficient for the Chinese population. Both scales contain 20 items and are judged on a scale of 1 to 4 to assess all statements (rarely, sometimes, frequently, or always). The total crude score ranges from 20 to 80 points and is then multiplied by 1.25 to obtain a standard scale. The cut-off standard score of SAS is 50 points, and a score ≥ 50 points indicates probable anxiety. Meanwhile, a standard SDS score ≥ 53 indicates positive depression screening. The higher the score, the higher the degree of anxiety or depression [28, 29]. The Cronbach’s alphas were 0.84 for SAS and 0.87 for SDS in the present study.

### Statistical analysis

Statistical analysis was performed using SPSS 26.0 (IBM SPSS, Chicago, IL, USA). The enumeration data are presented as the numbers (*n*) and percentages, and the measurement data are described as the means (*SD*). The data between groups were compared using Student’s *t*-test and the chi-square test. The single-sample *t*-test was used to compare the neurology staff’s scale scoring with Chinese norm scoring [30]. Multivariate logistic regression analysis was performed to evaluate the independent risk factors of probable anxiety and depression. Significant variables identified by bivariate analysis were then entered into the regression models. The results were expressed as the odds ratios (OR) with 95% confidence intervals (CIs). Two-tailed *P* values < 0.05 were considered to indicate statistical significance.

## Results

### Sociodemographic characteristics and attitudes of neurological doctors and nurses

The 612 neurology staff members included 317 doctors and 295 nurses. The two groups differed with respect to sex, age, education level, marital status, and occupational title. Most nurses were female, with only six male nurses responding. With age stratified, 20.1% of all subjects were above 40 years and 79.9% below or equal to 40 years, and nurses were younger than doctors, with 90.5% of nurses below 40 years old. The education levels were divided into undergraduate or lower and graduate or higher. The proportion of doctors with high education was higher than that of nurses (54.6% vs. 2.4%, respectively, *P* <  0.001). The number of single nurses was higher than that of single doctors. The proportion of senior occupational titles in doctors was higher than that in nurses (41.6% vs. 8.5%, respectively, *P* <  0.001). Among all participants, 210 (34.3%) agreed, 108 (17.6%) disagreed, and 294 (48.0%) were uncertain that the department of neurology was a high-risk place for COVID-19 (*P* = 0.002), and the proportion in agreement was higher for nurses than doctors. Only a few (10.9%) medical workers in the neurology department thought that the protective measures were adequate to prevent infection, with no significant difference between doctors and nurses. This may be due to the fear of infection, as some medical workers were not willing to treat or care for infected patients, and we found that the proportion of doctors expressing unwillingness was higher than nurses (*P* <  0.001) (Table 1).

**Table 1:** Demographic characteristics and attitudes toward COVID-19 of neurological doctors and nurses

Parameters	Total (***n*** = 612)	Doctors (***n*** = 317)	Nurses (***n*** = 295)	***P*** value
**Sex**				
Male	166 (27.1%)	160 (50.5%)	6 (2.0%)	< 0.001
Female	446 (72.9%)	157 (49.5%)	289 (98.0%)	
**Age**				
≤ 40 years	489 (79.9%)	222 (70.0%)	267 (90.5%)	< 0.001
> 40 years	123 (20.1%)	95 (30.0%)	28 (9.5%)	
**Education level**				
Undergraduate or lower	432 (70.6%)	144 (45.4%)	288 (97.6%)	< 0.001
Graduate or higher	180 (29.4%)	173 (54.6%)	7 (2.4%)	
**Marital status**				
Married	506 (82.7%)	277 (87.4%)	229 (77.6%)	0.001
Single	106 (17.3%)	40 (12.6%)	66 (22.4%)	
**Title**				
Senior	157 (25.7%)	132 (41.6%)	25 (8.5%)	< 0.001
Intermediate	208 (34.0%)	112 (35.3%)	96 (32.5%)	
Junior or lower	247 (40.4%)	73 23.0%)	174 (59.0%)	
**Do you think the department of neurology is a high-risk place for COVID-19?**				
Yes	210 (34.3%)	94 (29.7%)	116 (39.3%)	0.002
No	108 (17.6%)	71 (22.4%)	37 (12.5%)	
Uncertain	294 (48.0%)	152 (47.9%)	142 (48.1%)	
**Are your current precautions adequate to prevent infection?**				
Yes	67 (10.9%)	37 (11.7%)	30 (10.2%)	0.293
No	258 (42.2%)	141 (44.5%)	117 (39.7%)	
Uncertain	287 (46.9%)	139 (43.8%)	148 (50.2%)	
**Are you willing to treat or care for patients with COVID-19?**				
Yes	298 (48.7%)	142 (44.8%)	156 (52.9%)	< 0.001
No	100 (16.3%)	75 (23.7%)	25 (8.5%)	
Uncertain	214 (35.0%)	100 (31.5%)	114 (38.6%)	
Probable Anxiety	100 (16.3%)	40 (12.6%)	60 (20.3%)	0.010
Probable Depression	153 (25.0%)	64 (20.2%)	89 (30.2%)	0.004

*Abbreviations*: *COVID-19* Coronavirus Disease 2019

### Anxiety and depression of participants

The overall prevalence of probable anxiety and depression was 16.3 and 25.0%, respectively. Nurses had a higher proportion of probable anxiety (20.3% vs. 12.6%, respectively, *P* = 0.010) and depression (30.2% vs. 20.2%, *P* = 0.004) than doctors (Table 1). The average SAS standard score of participants was higher than the Chinese national norms (41.33 ± 8.98 vs. 33.8 ± 5.9, *P* <  0.001). The average SDS standard score of participants was 41.96 ± 11.46, which showed no significant difference when compared with the Chinese national norms (41.85 ± 10.57).

The prevalence of probable anxiety and depression among medical staff by basic characteristic is shown in Table 2. Female medical workers had a significantly higher proportion of anxiety than males (18.4% vs. 10.8%, *P* = 0.025). Probable anxiety and depression were more prevalent among neurological nurses, younger workers (≤ 40 years), and medical staff with junior titles (*P* <  0.05, respectively). We also found that workers who thought that the protective measures were not sufficient to prevent infection were more likely to report probable anxiety (*P* = 0.009).

**Table 2:** Prevalence of probable anxiety and depression among neurological workers by different characteristics

Parameters	Probable Anxiety	***P*** value	Probable Depression	***P*** value
**Sex**				
Male	18 (10.8%)	0.025	34 (20.5%)	0.115
Female	82 (18.4%)		119 (26.7%)	
**Age**				
≤ 40 years	88 (18.0%)	0.027	138 (28.2%)	< 0.001
> 40 years	12 (9.8%)		15 (12.2%)	
**Education level**				
Undergraduate or lower	77 (17.8%)	0.124	116 (26.9%)	0.101
Graduate or higher	23 (12.8%)		37 (20.6%)	
**Marital status**				
Married	86 (17.0%)	0.337	125 (24.7%)	0.711
Single	14 (13.2%)		28 (26.4%)	
**Occupation**				
Doctor	40 (12.6%)	0.012	64 (20.2%)	0.004
Nurse	60 (20.3%)		89 (30.2%)	
**Title**				
Senior	18 (11.5%)	0.041	25 (15.9%)	0.006
Intermediate	31 (14.9%)		54 (26.0%)	
Junior or lower	51 (20.6%)		74 (30.0%)	
**Do you think the department of neurology is a high-risk place for COVID-19?**				
Yes	40 (19.0%)	0.126	61 (29.0%)	0.193
No	11 (10.2%)		22 (20.4%)	
Uncertain	49 (16.7%)		70 (23.8%)	
**Are your current precautions adequate to prevent infection?**				
Yes	8 (11.9%)	0.009	17 (25.4%)	0.537
No	56 (21.7%)		70 (27.1%)	
Uncertain	36 (12.5%)		66 (23.0%)	
**Are you willing to treat or care for patients with COVID-19?**				
Yes	44 (14.8%)	0.569	67 (22.5%)	0.300
No	17 (17.0%)		25 (25.0%)	
Uncertain	39 (18.2%)		61 (28.5%)	

Abbreviations: COVID-19: Coronavirus Disease 2019

In further analysis, we used multivariate logistic regression analysis to identify the independent factors of probable anxiety and depression. Variables showing *P* <  0.05 in the bivariate analysis were selected for entry into the multivariate logistic regression analysis. The results of the multivariate analysis (Table 3) indicated that medical workers who disagreed that the current protective measures were adequate to prevent infection were significantly more likely to have probable anxiety (OR = 1.980, 95% CI: 1.241–3.160, *P* = 0.004). Probable depression was significantly associated with young age (≤ 40) (OR = 2.293, 95% CI: 1.137–4.623, *P* = 0.020).

**Table 3:** Multivariate Logistic regression analysis of probable anxiety and depression in neurological workers

	Probable Anxiety			Probable Depression		
Variables	***P*** value	OR	95% CI for OR	***P*** value	OR	95% CI for OR
**Sex**						
Male	Reference			Reference		
Female	0.436	1.300	0.672–2.514	0.630	0.876	0.511–1.501
**Age**						
> 40 years	Reference			Reference		
≤ 40 years	0.365	1.446	0.651–3.212	0.020	2.293	1.137–4.623
**Education level**						
Undergraduate or lower	Reference			Reference		
Graduate or higher	0.859	0.943	0.494–1.801	0.834	0.944	0.551–1.617
**Marital status**						
Married	Reference			Reference		
Single	0.066	0.543	0.283–1.041	0.571	0.861	0.514–1.444
**Occupation**						
Doctor	Reference			Reference		
Nurse	0.395	1.326	0.692–2.544	0.183	1.463	0.836–2.560
**Title**						
Junior or lower	Reference			Reference		
Intermediate	0.925	1.036	0.496–2.161	0.622	1.170	0.627–2.181
Senior	0.241	1.595	0.731–3.478	0.465	1.285	0.656–2.518
**Are your current precautions adequate to prevent infection?**						
Yes	0.977	1.013	0.443–2.316	0.568	1.201	0.641–2.249
No	0.004	1.980	1.241–3.160	0.229	1.276	0.858–2.518
Uncertain	Reference			Reference		

## Discussion

This is the first study to report the prevalence of probable anxiety and depression among neurological doctors and nurses in Hunan Province during the COVID-19 outbreak. Our study found that probable anxiety and depression were more prevalent among neurological nurses than doctors. The shortage of protective equipment and young age were the main factors influencing anxiety and depression of neurological healthcare workers, respectively.

In dealing with this large-scale public health emergency, healthcare workers experienced both physical and psychological pressure. A retrospective clinical study of 138 hospitalized patients from Zhongnan Hospital of Wuhan University found that novel coronavirus pneumonia caused by hospital-related transmission was common, as 40 (29%) healthcare workers were presumed to have been infected in hospitals. Of these patients with nosocomial infections, 31 (77.5%) were from the general wards, seven (17.5%) were from the emergency department, and two (5%) were from the intensive care unit (ICU) [4]. What is worse, at least 3000 medical workers across the Chinese mainland have been infected with the novel coronavirus during the nationwide outbreak, according to epidemiological characteristics of the 2019 COVID-19 outbreak in China [1]. As the number of infected medical staff members increases, medical workers have been experiencing psychological disorders, such as anxiety, depression, phobia and sleep disturbances [31–33]. In our study, the overall prevalence of probable anxiety and depression was 16.3 and 25.0%, respectively. According to one recent meta- analysis, the pooled prevalence among healthcare workers was 16.47% for anxiety as assessed by SAS and 32.81% for depression as assessed by SDS [34]. In another meta-analysis, the pooled prevalence of anxiety and depression among healthcare workers was 26% (18–34%) and 25% (17–33%), respectively, but the prevalence of depression in China could be as high as 51% (48–53%) [35]. The prevalence of probable anxiety and depression was relatively lower than that for medical workers in the frontline in China, which could be explained by the heavy pressure of work and high infectious potential in their workplaces. Depression was more common than anxiety in this study, which is consistent with data reported in other studies and in the above two meta-analyses. The decline in social activities and the risk of contracting COVID-19, social isolation, and spending more time watching COVID-19-related news, which is common during lockdown, could be the main risk factors of depression [35].

This SARS-like coronavirus has the ability to use the cell entry receptor angiotensin-converting enzyme 2 (ACE2) and replicate in human cells of multiple human organs, including the nervous system [36, 37], leading to abnormally high blood pressure and increasing the risk of cerebral hemorrhage. In China, the presence of 2019-nCoV in the cerebrospinal fluid was confirmed by gene sequencing of a 56-year-old patient with COVID-19 in Beijing [38]. The neurological symptoms of patients with COVID-19 have been described in some studies. Some patients were admitted to the hospital with symptoms of sudden slurred speech, limb paralysis, headache, epilepsy, or confusion [17, 21]. As general wards far from the front line, departments of neurology are also considered high-risk. In our study, 210 (34.3%) medical workers thought that the department of neurology was a high-risk place for COVID-19; the proportion of doctors holding this attitude was greater than that of nurses. Only 67 medical workers agreed that the current protective measures were adequate to prevent infection, accounting for 10.9% of the total. Volunteer medical workers have been recruited from other departments to assist frontline medical personnel. Many neurological staff members are willing to treat or care for infected patients, and the proportion holding this attitude was also higher among nurses than doctors.

We found that female medical workers were more likely to develop probable anxiety than males. Nurses and younger workers and those who had lower occupational titles were more prone to both anxiety and depression. Our findings are generally consistent with other studies on COVID-19 and previous studies on SARS in 2003 [39–42], which reported that women and nurses reported more severe symptoms of anxiety and distress. Furthermore, a recent meta-analysis found that women and nurses were more vulnerable to stress [35, 39]. Nurses play a critically important role in the battle against COVID-19, and they have a higher risk of infection due to their close contact with patients during nursing work. In our study, almost half of the doctors were women, and 98% of the nurses were women. Women may be more prone to anxiety, possibly due to the high risk of infection, heavy pressure from family, and effects of female hormones [42]. Moreover, 59% of all nurses had junior titles or below, indicating less work experience [39]. Similarly, medical workers aged below 40 years and having lower occupational titles faced mental health disorders of anxiety and depression, probably due to insufficient experience in dealing with this public health emergency, similar to the findings in Taiwan during the SARS outbreak [43]. In our study, sex was not correlated with probable depression, and another study also indicated that female gender was a risk factor for anxiety but not depression [44]. In the logistic regression analysis, occupation and sex were not independently associated with mental health outcome. Different studies have had different conclusions, and more studies about the mental health of healthcare workers with larger sample sizes are needed.

The fear of uncertainty of the coronavirus transmission routes and the dissemination of negative information about the infection of medical staff have resulted in high levels of anxiety among medical workers. We found that the shortage of protective equipment was independently associated with anxiety. Younger age was an independent risk factor for depression. Since the outbreak, there has been a shortage of medical protective supplies, such as medical protective clothing, N95 masks, medical masks, protective masks, and goggles, which are urgently needed for the prevention and control of the epidemic and have severely threatened the safety of health care workers. During the COVID-19 outbreak, primary protection measures were recommended in the neurology clinic and wards, while secondary protection measures were used for high-risk exposed personnel when dealing with suspected patients to alleviate the shortage of supplies [21]. Nonetheless, primary protective measures like surgical masks remained in seriously short supply in neurology departments. It is difficult for neurological workers to differentiate and screen patients with manifestations of neurological systems that may be initial symptoms without fever or pulmonary disorders, which may lead to inadvertent exposure of medical staff to the virus. Other sources of stress reported by another study included reduced accessibility to formal psychological support, less up-to-date and accurate health information, and less intensive training on personal protective equipment and infection control measures [45].

Multifaceted interventions should also be undertaken to relieve anxiety and depression among medical workers in neurology departments. First, preliminary checks and differential diagnoses of suspected cases should be firmly implemented to ensure safety at the front line. Second, employees in departments of neurology should acquire in-depth knowledge of infection prevention to improve compliance with hand disinfection and personal protective measures. Third, with the opening of outpatient appointments, hospitals should also pay attention to medical workers out of the frontline and provide adequate protective equipment to reduce their risk of infection. Fourth, we can learn from the experiences of the Second Xiangya Hospital in Hunan Province and establish such resources as online courses and psychological assistance hotline teams to guide medical workers in dealing with common mental health problems and various group activities to help staff release stress [23]. Workers with psychological disorders can also use online psychological self-help intervention systems to reduce symptoms of anxiety and depression [31] and apply a virtual platform characterized by mindfulness-based therapy to develop psychological resilience [46]. Fifth, our government should strengthen support for and safeguard the legitimate rights and interests of medical workers during epidemic control and in the future.

This study had several limitations. First, the participants were all from Hunan Province, the province near Hubei Province, limiting the generalization of our findings to other studies. Second, it was limited by its use of the SAS and SDS to measure symptoms of anxiety and depression, which was different from a clinical diagnosis and did not measure severe psychiatric symptoms, such as suicidal ideation or psychotic experience [33]. Third, the study was cross-sectional, and no cause-effect relationship can thus be established between the attitude toward COVID-19 and mental health disorders. Fourth, due to the limited time for designing the questionnaire, the attitude toward COVID-19 only included three simple questions, lacking multi-dimensional measures. Fifth, the snowballing sampling strategy was not ideal for estimating prevalence due to selection bias, and the small sample size in our study reduced its reliability. Sixth, the electronic questionnaire was taken and well-accepted mainly by young people, most healthcare workers in our study were young, which may be different from our target population (all neurological clinicians in the region) and it might cause sampling bias to some extent. Furthermore, influencing factors, such as history of mental health conditions or having high risk individuals in the household, were not included in this study.

## Conclusion

During the fight against COVID-19 in Hunan Province, the shortage of protective equipment has led to probable anxiety, and young age has led to probable depression among medical staff in departments of neurology. Much more attention should be paid to medical workers out of the frontline, including providing necessary protective equipment and psychological assistance.

## Data Availability

Generated Statement: The datasets generated for this study are available on request to the corresponding author.

## Abbreviations

COVID-19: Coronavirus Disease 2019
SAS: Zung Self-Rating Anxiety Scale
SDS: Zung Self-Rating Depression Scale
2019-nCoV: 2019 novel coronavirus
SARS-CoV: Severe acute respiratory syndrome coronavirus
MERS-CoV: Middle East respiratory syndrome coronavirus
CNS: Central nervous system
ACE2: Angiotensin-converting enzyme 2

## References

[1] Epidemiology Working Group for NCIP Epidemic Response, Chinese Center for Disease Control and Prevention. The epidemiological characteristics of an outbreak of 2019 novel coronavirus diseases (COVID-19) in China. Zhonghua Liu Xing Bing Xue Za Zhi. 2020;41(2):145–51. 10.3760/cma.j.issn.0254-6450.2020.02.003. 10.3760/cma.j.issn.0254-6450.2020.02.003 32064853

[2] Mahase E (2020). Coronavirus covid-19 has killed more people than SARS and MERS combined, despite lower case fatality rate. BMJ (Clin Res Ed).

[3] Peeri NC, Shrestha N, Rahman MS, Zaki R, Tan Z, Bibi S, Baghbanzadeh M, Aghamohammadi N, Zhang W, Haque U (2020). The SARS, MERS and novel coronavirus (COVID-19) epidemics, the newest and biggest global health threats: what lessons have we learned?. Int J Epidemiol.

[4] Wang D, Hu B, Hu C, Zhu F, Liu X, Zhang J, Wang B, Xiang H, Cheng Z, Xiong Y (2020). Clinical characteristics of 138 hospitalized patients with 2019 novel coronavirus-infected pneumonia in Wuhan, China. Jama.

[5] Huang C, Wang Y, Li X, Ren L, Zhao J, Hu Y, Zhang L, Fan G, Xu J, Gu X (2020). Clinical features of patients infected with 2019 novel coronavirus in Wuhan, China. Lancet.

[6] Chen N, Zhou M, Dong X, Qu J, Gong F, Han Y, Qiu Y, Wang J, Liu Y, Wei Y (2020). Epidemiological and clinical characteristics of 99 cases of 2019 novel coronavirus pneumonia in Wuhan, China: a descriptive study. Lancet.

[7] Yang Y, Peng F, Wang R, Yange M, Guan K, Jiang T, Xu G, Sun J, Chang C (2020). The deadly coronaviruses: the 2003 SARS pandemic and the 2020 novel coronavirus epidemic in China. J Autoimmun.

[8] Kui L, Fang YY, Deng Y, Liu W, Wang MF, Ma JP, Xiao W, Wang YN, Zhong MH, Li CH (2020). Clinical characteristics of novel coronavirus cases in tertiary hospitals in Hubei Province. Chin Med J.

[9] Wang XF, Yuan J, Zheng YJ, Chen J, Bao YM, Wang YR, Wang LF, Li H, Zeng JX, Zhang YH (2020). Clinical and epidemiological characteristics of 34 children with 2019 novel coronavirus infection in Shenzhen. Zhonghua Er Ke Za Zh.

[10] An update on the epidemiological characteristics of novel coronavirus pneumoniaCOVID-19 (2020). Zhonghua Liu Xing Bing Xue Za Zhi.

[11] Bai Y, Yao L, Wei T, Tian F, Jin DY, Chen L, Wang M (2020). Presumed asymptomatic carrier transmission of COVID-19. Jama.

[12] Phan LT, Nguyen TV, Luong QC, Nguyen TV, Nguyen HT, Le HQ, Nguyen TT, Cao TM, Pham QD (2020). Importation and human-to-human transmission of a novel coronavirus in Vietnam. N Engl J Med.

[13] van Doremalen N, Bushmaker T, Morris DH, Holbrook MG, Gamble A, Williamson BN, Tamin A, Harcourt JL, Thornburg NJ, Gerber SI (2020). Aerosol and surface stability of SARS-CoV-2 as compared with SARS-CoV-1. N Engl J Med.

[14] Wang C, Pan R, Wan X, Tan Y, Xu L, Ho CS, Ho RC (2020). Immediate psychological responses and associated factors during the initial stage of the 2019 coronavirus disease (COVID-19) epidemic among the general population in China. Int J Environ Res Public Health.

[15] Wang MW, Zhou MY, Ji GH, Ye L, Cheng YR, Feng ZH, Chen J (2020). Mask crisis during the COVID-19 outbreak. Eur Rev Med Pharmacol Sci.

[16] Desforges M, Le Coupanec A, Dubeau P, Bourgouin A, Lajoie L, Dube M, Talbot PJ (2019). Human coronaviruses and other respiratory viruses: underestimated opportunistic pathogens of the central nervous system?. Viruses.

[17] Mao L, Jin H, Wang M, Hu Y, Chen S, He Q, Chang J, Hong C, Zhou Y, Wang D (2020). Neurologic manifestations of hospitalized patients with coronavirus disease 2019 in Wuhan, China. JAMA Neurol.

[18] Zhou F, Yu T, Du R, Fan G, Liu Y, Liu Z, Xiang J, Wang Y, Song B, Gu X (2020). Clinical course and risk factors for mortality of adult inpatients with COVID-19 in Wuhan, China: a retrospective cohort study. Lancet (London, England).

[19] Oxley TJ, Mocco J, Majidi S, Kellner CP, Shoirah H, Singh IP, De Leacy RA, Shigematsu T, Ladner TR, Yaeger KA (2020). Large-vessel stroke as a presenting feature of Covid-19 in the young. N Engl J Med.

[20] Avula A, Nalleballe K, Narula N, Sapozhnikov S, Dandu V, Toom S, Glaser A, Elsayegh D (2020). COVID-19 presenting as stroke. Brain Behav Immun.

[21] Jin H, Hong C, Chen S, Zhou Y, Wang Y, Mao L, Li Y, He Q, Li M, Su Y (2020). Consensus for prevention and management of coronavirus disease 2019 (COVID-19) for neurologists. Stroke Vasc Neurol.

[22] Lu YC, Shu BC, Chang YY, Lung FW (2006). The mental health of hospital workers dealing with severe acute respiratory syndrome. Psychother Psychosom.

[23] Chen Q, Liang M, Li Y, Guo J, Fei D, Wang L, He L, Sheng C, Cai Y, Li X (2020). Mental health care for medical staff in China during the COVID-19 outbreak. Lancet Psychiatry.

[24] Chua SE, Cheung V, Cheung C, McAlonan GM, Wong JW, Cheung EP, Chan MT, Wong MM, Tang SW, Choy KM (2004). Psychological effects of the SARS outbreak in Hong Kong on high-risk health care workers. Can J Psychiatry.

[25] Chong MY, Wang WC, Hsieh WC, Lee CY, Chiu NM, Yeh WC, Huang OL, Wen JK, Chen CL (2004). Psychological impact of severe acute respiratory syndrome on health workers in a tertiary hospital. Br J Psychiatry.

[26] Hao F, Tan W, Jiang L, Zhang L, Zhao X, Zou Y, Hu Y, Luo X, Jiang X, McIntyre RS (2020). Do psychiatric patients experience more psychiatric symptoms during COVID-19 pandemic and lockdown? A case-control study with service and research implications for immunopsychiatry. Brain Behav Immun.

[27] Zhang WR, Wang K, Yin L, Zhao WF, Xue Q, Peng M, Min BQ, Tian Q, Leng HX, Du JL, et al. Mental Health and Psychosocial Problems of Medical Health Workers during the COVID-19 Epidemic in China. Psychother Psychosom. 2020;89(4):242–50. 10.1159/000507639 PMC7206349 32272480

[28] Zung WW (1971). A rating instrument for anxiety disorders. Psychosomatics.

[29] Zung WW (1965). A self-rating depression scale. Arch Gen Psychiatry.

[30] Shen LL, Lao LM, Jiang SF, Yang H, Ren LM, Ying DG, Zhu SZ (2012). A survey of anxiety and depression symptoms among primary-care physicians in China. Int J Psychiatry Med.

[31] Liu S, Yang L, Zhang C, Xiang YT, Liu Z, Hu S, Zhang B (2020). Online mental health services in China during the COVID-19 outbreak. Lancet Psychiatry.

[32] Chew NWS, Lee GKH, Tan BYQ, Jing M, Goh Y, Ngiam NJH, Yeo LLL, Ahmad A, Ahmed Khan F, Napolean Shanmugam G (2020). A multinational, multicentre study on the psychological outcomes and associated physical symptoms amongst healthcare workers during COVID-19 outbreak. Brain Behav Immun.

[33] Tan W, Hao F, McIntyre RS, Jiang L, Jiang X, Zhang L, Zhao X, Zou Y, Hu Y, Luo X (2020). Is returning to work during the COVID-19 pandemic stressful? A study on immediate mental health status and psychoneuroimmunity prevention measures of Chinese workforce. Brain Behav Immun.

[34] Pappa S, Ntella V, Giannakas T, Giannakoulis VG, Papoutsi E, Katsaounou P (2020). Prevalence of depression, anxiety, and insomnia among healthcare workers during the COVID-19 pandemic: a systematic review and meta-analysis. Brain Behav Immun.

[35] Luo M, Guo L, Yu M, Jiang W, Wang H (2020). The psychological and mental impact of coronavirus disease 2019 (COVID-19) on medical staff and general public - a systematic review and meta-analysis. Psychiatry Res.

[36] Zhou P, Yang XL, Wang XG, Hu B, Zhang L, Zhang W, Si HR, Zhu Y, Li B, Huang CL (2020). A pneumonia outbreak associated with a new coronavirus of probable bat origin. Nature.

[37] Tian X, Li C, Huang A, Xia S, Lu S, Shi Z, Lu L, Jiang S, Yang Z, Wu Y (2020). Potent binding of 2019 novel coronavirus spike protein by a SARS coronavirus-specific human monoclonal antibody. Emerg Microbes Infect.

[38] Zhou L, Zhang M, Wang J, Gao J. Sars-Cov-2: underestimated damage to nervous system. Travel Med Infect Dis. 2020:101642. 10.1016/j.tmaid.2020.101642. 10.1016/j.tmaid.2020.101642 PMC7269702 32220634

[39] Lai J, Ma S, Wang Y, Cai Z, Hu J, Wei N, Wu J, Du H, Chen T, Li R (2020). Factors associated with mental health outcomes among health care workers exposed to coronavirus disease 2019. JAMA Netw Open.

[40] Chan S (2003). Nurses fighting against severe acute respiratory syndrome (SARS) in Hong Kong. J Nurs Scholarsh.

[41] Wong TW, Yau JK, Chan CL, Kwong RS, Ho SM, Lau CC, Lau FL, Lit CH (2005). The psychological impact of severe acute respiratory syndrome outbreak on healthcare workers in emergency departments and how they cope. Eur J Emerg Med.

[42] Elbay RY, Kurtulmuş A, Arpacıoğlu S, Karadere E (2020). Depression, anxiety, stress levels of physicians and associated factors in Covid-19 pandemics. Psychiatry Res.

[43] Su TP, Lien TC, Yang CY, Su YL, Wang JH, Tsai SL, Yin JC (2007). Prevalence of psychiatric morbidity and psychological adaptation of the nurses in a structured SARS caring unit during outbreak: a prospective and periodic assessment study in Taiwan. J Psychiatr Res.

[44] Özdin S, Bayrak Özdin Ş. Levels and predictors of anxiety, depression and health anxiety during COVID-19 pandemic in Turkish society: The importance of gender. The International journal of social psychiatry. 2020;66(5):504–11. 10.1177/0020764020927051 PMC7405629 32380879

[45] Tan BYQ, Chew NWS, Lee GKH, Jing M, Goh Y, Yeo LLL, Zhang K, Chin HK, Ahmad A, Khan FA (2020). Psychological impact of the COVID-19 pandemic on health Care Workers in Singapore. Ann Intern Med.

[46] Ho CS, Chee CY, Ho RC (2020). Mental health strategies to combat the psychological impact of COVID-19 beyond paranoia and panic. Ann Acad Med Singap.

